# Scrub Typhus Outbreak, Northern Thailand, 2006–2007

**DOI:** 10.3201/eid1905.121445

**Published:** 2013-05

**Authors:** Wuttikon Rodkvamtook, Jariyanart Gaywee, Suparat Kanjanavanit, Toon Ruangareerate, Allen L. Richards, Noppadon Sangjun, Pimmada Jeamwattanalert, Narongrid Sirisopana

**Affiliations:** Armed Forces Research Institute of Medical Science, Bangkok, Thailand (W. Rodkvamtook, J. Gaywee, T. Ruangareerate, N. Sangjun, P. Jeamwattanalort, N. Sirisopana);; Nakhornping Hospital, Chiang Mai, Thailand (S. Kanjanavanit);; Naval Medical Research Center, Silver Spring, Maryland, USA (A. L. Richards).

**Keywords:** scrub typhus, Orientia tsutsugamushi, outbreak investigation, parasites, trombiculid mites, chiggers, small mammals, mammal reservoirs, rodents, transmission vector, Thailand, zoonoses, bacteria

## Abstract

During a scrub typhus outbreak investigation in Thailand, 4 isolates of *O. tsutsugamushi* were obtained and established in culture. Phylogenetic analysis based on the 56-kDa type-specific antigen gene demonstrated that the isolates fell into 4 genetic clusters, 3 of which had been previously reported and 1 that represents a new genotype.

Scrub typhus is a febrile disease endemic to the Asia–Australia–Pacific region, where ≈1 million cases occur annually (*1*). The causative agent of scrub typhus in this region is the gram-negative obligate intracellular bacterium *Orientia tsutsugamushi* (*2*). The bacterium maintains itself in trombiculid mites, and small mammals serve as reservoir hosts in the natural life cycle of the mites. Chiggers, the larval stage of mites, act as the transmission vector for *O. tsutsugamushi* (*1*). Humans and small animals become infected following the bite of chiggers harboring *O. tsutsugamushi*. After an incubation period of 7–14 days, high fever, chills, headache, rash, and an eschar usually develop in infected persons (*3*).

Scrub typhus is endemic to northern Thailand, especially Chiang Mai Province, where >200 cases are reported each year (*4*). During June 2006–May 2007, a total of 142 febrile children with clinically suspected scrub typhus were admitted to Nakornping Hospital in the city of Chiang Mai. Serologic and molecular laboratory test results showed that 65 of the children were positive for *O. tsutsugamushi*. Among the 142 hospitalized children, 30 were Hmong hill tribe people living in Ban Pongyeang, a village in the mountain area located north of the Chiang Mai. Laboratory testing also confirmed that 26 of the 30 Hmong children had scrub typhus.

To better characterize the specific strain(s) of *O. tsutsugamushi* present in the area and to determine how the agent(s) is transmitted to humans, we genetically typed *O. tsutsugamushi* obtained from these 26 children and small mammals. The Royal Thai Army Medical Department Ethical Committee approved all procedures (protocol S014q/45). Small mammals were handled according to guidelines in the Guide for the Care and Use of Laboratory Animals (National Institutes of Health publication no. 85–23, revised 1985).

## The Study

We obtained clinical information and blood samples from 26 scrub typhus–infected children from Ban Pongyeang after their parents gave informed consent. Blood specimens were stored in liquid nitrogen and shipped on dry ice to the Armed Forces Research Institute of Medical Sciences in Bangkok, Thailand, for serologic testing, genetic characterization, and isolation of *O. tsutsugamushi.*

We assessed serum samples for the presence of antibodies against *O. tsutsugamushi* by using an indirect fluorescence antibody assay (*5*) with an in-house antigen preparation from propagated *O. tsutsugamushi* Karp, Kato, and Gilliam strains. Single specimens with an IgM or IgG titer >400 were considered positive; paired specimens were considered positive if they showed seroconversion or a >4-fold rise in titer (*6*). To genetically characterize *O. tsutsugamushi*, we amplified a fragment of the 56-kDa type-specific antigen gene from patients’ blood genomic DNA by using a modified nested PCR procedure as described (*7*). A newly designed forward primer (F584, 5′-CAA TGT CTG CGT TGT CGT TGC-3′) was used with the previously reported reverse primers RTS9 and RTS8 (*7*). The expected 693-bp products were purified, directly sequenced, and aligned according to ClustalW algorithm (www.clustal.org/). Using PAUP 4.0b10 software and maximum parsimony methods, we generated phylogenetic relationships (*8*). *O. tsutsugamushi* was isolated by using animal inoculation and L-929 mouse fibroblast cell culture techniques as described (*9*).

**Figure 1:**
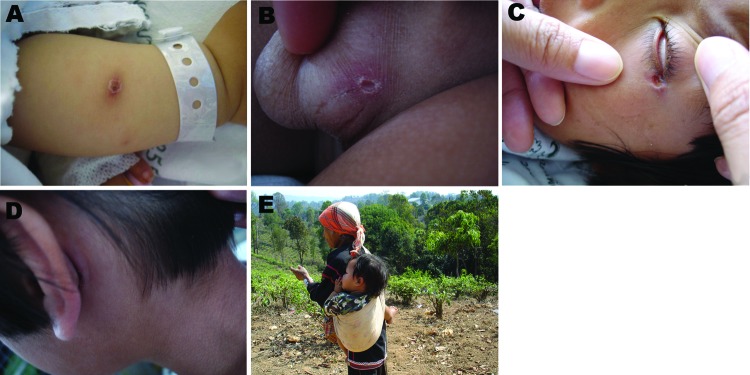
Eschars in different body areas of children with scrub typhus (A–D) and a child carried on his mother’s back during work (E), Ban Pongyeang, Thailand.

Patient clinical information and laboratory test results are shown in the Technical Appendix. The patients’ ages ranged from 11 months to 13 years. Common signs and symptoms of illness were fever (100.0%), chills (73.1%), eschar (73.1%), headache (57.7%), and rash (23.1%) (Technical Appendix; Figure 1). Of the 26 patients, 23 showed seroreactivity to *O. tsutsugamushi* antigens; PCR confirmed the presence of *O. tsutsugamushi* DNA in 24/26 patients (Technical Appendix). Two *O. tsutsugamushi* isolates (PYH1 and PYH4) were successfully established from EDTA whole blood samples of 7 patients (Technical Appendix). Patient histories revealed that the infected children commonly played in grassland, woods, and rice fields. Cases also occurred in infants who were carried on their mother’s back during work in those areas (Figure 1E). In addition, the opportunity to become infected was increased by frequent exposure to vector mites living in vegetation-rich areas.

To investigate *O. tsutsugamushi* transmission, we trapped small mammals from different terrains in Ban Pongyeang, identified them to species level, and collected tissue specimens (whole blood, liver, and spleen). The specimens were kept in liquid nitrogen and delivered to the Armed Forces Research Institute of Medical Sciences for laboratory testing. Chiggers were removed from captured mammals and stored in 70% ethanol. The chiggers were slide-mounted and identified to species by using a microscope.

A total of 55 small wild mammals were captured from different terrains in Ban Pongyeang, such as grass, rice, and banana fields and areas with shrubs and woods. The collected animals included greater bandicoot rats (*Bandicota indica*), Savile's bandicoot rats (*B. savilei*), black rats (*Rattus rattus*), small white-tooth rats (*R. berdmorei*), Polynesian rats (*R. exulans*), Berdmore's ground squirrels (*Menetes berdmorei*), a common tree shrew (*Tupaia glis*), and a small Asian mongoose (*Herpestes javanicus*) (Table 1). 

**Table 1:** Chigger infestation and *Orientia tsutsugamushi* infection in small mammals captured in Ban Pongyeang, northern Thailand, 2006–2007*

Rodent family, genus species	No. animals captured	No. (%) animals infested with chiggers	No. chiggers collected (mean no./animal)	No. (%) animals with *O. tsutsugamushi* infection	No. (%) *O. tsutsugamushi* isolates obtained
Muridae					
* Bandicota indica*	15	15 (100.0)	951 (63.4)	15 (100.0)	2 (22.2)
* B. savilei*	12	12 (100.0)	699 (58.2)	9 (75.0)	0
* Rattus rattus*	15	8 (55.6)	320 (21.3)	8 (53.3)	0
* R. exulans*	3	1 (50.0)	40 (5.0)	1 (33.3)	0
* R. berdmorei*	6	4 (66.6)	168 (28.0)	3 (50.0)	0
Viverridae, *Herpestes javanicus*	1	1 (100.0)	7 (7.0)	ND	NA
Sciuridae, *Menetes berdmorei*	2	2 (100.0)	56 (28.0)	ND	NA
Tupaiidae, *Tupaia glis*	1	1 (100.0)	41 (41.0)	ND	NA
Total	55	45 (81.8)	2,277 (44.4)	36 (65.5)	2 (3.6)

*ND, not done; NA, not applicable.

Forty-five (81.8%) mammals were infested with a total of 2,277 chiggers (Table 1). A *B. indica* and a *B. savilei* rat had the highest chigger densities. Collected chiggers were classified to 4 species: *Leptotrombidium deliense* (47.6%; a well-known vector of scrub typhus), *Gahrliepia (Walchia) rustica* (35.1%), *G. (Schoengastiella) ligula* (14.6%), and *Ascoschoengastia* spp. (2.7%) (Table 2). 

**Table 2:** Species of chiggers collected from small mammals, Ban Pongyeang, northern Thailand, 2006–2007.

Host species	No. (%) chiggers				Total
	*Leptotrombidium deliense *	*Gahrliepia (Walchia) rustica*	*Gahrliepia (Schoengastiella) ligula*	*Ascoschoengastia* spp.	
*Bandicota indica*	471 (49.5)	324 (34.1)	131 (13.8)	25 (2.6)	951
*Bandicota savilei*	354 (50.7)	223 (31.9)	105 (15.0)	17 (2.4)	699
*Rattus rattus*	125 (39.1)	119 (37.2)	56 (17.4)	20 (6.3)	320
*Rattus exulans*	28 (70.0)	12 (30.0)	0	0	40
*Rattus berdmorei*	52 (31.0)	80 (47.6)	31 (18.5)	5 (2.9)	168
*Tupaia glis*	15 (36.6)	17 (41.5)	9 (21.9)	0	41
*Menetes berdmorei*	32 (57.1)	24 (42.9)	0	0	56
*Herpestes javanicus*	7 (100.0)	0	0	0	7
Total	1,084 (47.6)	799 (35.1)	332 (14.6)	62 (2.7)	2,277

Thirty-six (65.5%) of 51 animals tested were seroreactive to *O. tsutsugamushi* (Table 1). Compared with the other animals, a higher percentage (100%) of *B. indica* rats had *O. tsutsugamushi* infections, indicating that this species might serve as a reservoir host for the bacterium (Table 1). Because of limitations of commercial secondary antibodies, we could not perform indirect fluorescence antibody assays for the captured *T. glis* shrew (1), *M. berdmorei* ground squirrels (2), and *H. javanicus* mongoose (1). Two *O. tsutsugamushi* isolates (PYA5 and PYA6) were established from livers and spleens of 2 *B. indica* rats (Table 1). Together, the high prevalence of *O. tsutsugamushi*–seroreactive small mammals and the presence of infested scrub typhus–specific arthropod vectors indicate that scrub typhus is endemic to the Ban Pongyeang area.

**Figure 2:**
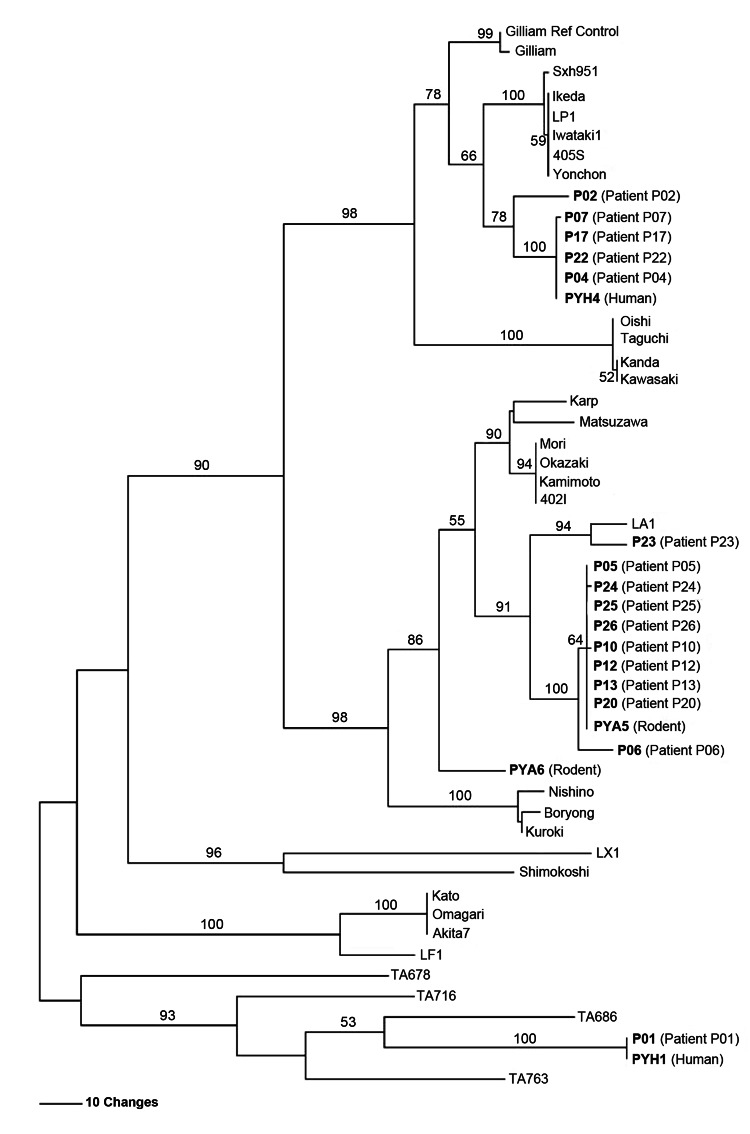
Maximum parsimony phylogenetic tree of *Orientia tsutsugamushi* based on partial 56-kDa type-specific antigen gene sequences, demonstrating the relationships among *O. tsutsugamushi* isolates from Thailand and strains causing scrub typhus in humans in Ban Pongyeang, Thailand, and reference (ref) strains. The tree was midpoint rooted. Bootstrap values >50% are labeled over branches (1,000 replicates). Isolates from Thailand are in **boldface**. The tree was generated by using heuristic search with random stepwise addition (10 replicates). Scale bar indicates nucleotide substitutions per site.

*O. tsutsugamushi* obtained from the infected children and small mammals was characterized on the basis of *Orientia* spp.–specific 56-kDa gene fragments. Multiple alignment and phylogenetic analysis demonstrated that the 4 *O. tsutsugamushi* isolates from Ban Pongyeang fell into 4 clusters. Sequences for 3 of the isolates clustered with Gilliam, LA, and TA, 3 genotypes that are commonly found in Southeast Asia (*10*,*11*); the sequence of the fourth isolate presented as a divergent distinct genotype (Figure 2). Most of the children were infected with a strain genetically similar to the LA cluster (Figure 2). Moreover, this major pathogenic strain was recovered from *B. indica* bandicoot rats (isolate PYA5), the most commonly found rats in the village and the small mammals with the highest densities of *L*. *deliense* chiggers. These findings indicate possible transmission between animals and humans. Many studies have demonstrated that chiggers can acquire *O. tsutsugamushi* during the feeding process (*12*–*15*). Therefore, rodents could play a critical role as reservoir hosts for *O. tsutsugamushi* and for feeding vector mites, causing widespread distribution of *O. tsutsugamushi* in Ban Pongyeang.

## Conclusions

Investigation of scrub typhus in Ban Pongyeang, northern Thailand, demonstrated *O. tsutsugamushi* infection in children and rodent hosts, and it demonstrated the potential for transmission between small mammal reservoirs and humans. Campaigns concerning protection from scrub typhus should be established in areas where *O. tsutsugamushi* is endemic, and local medical clinics should be made aware of the campaigns. Specific plans for protecting against/preventing *O. tsutsugamushi* transmission are crucially needed to prevent scrub typhus infection in humans.

Technical Appendix Clinical manifestations and laboratory test results for 26 scrub typhus–infected children from Ban Pongyeang, Thailand, who were hospitalized during June 2006–May 2007.

## References

[1] Rosenberg R. Drug-resistant scrub typhus: paradigm and paradox. Parasitol Today. 1997;13:131–2. 10.1016/S0169-4758(97)01020-X 15275098

[2] Tamura A, Ohashi N, Urakami H, Miyamura S. Classification of *Rickettsia tsutsugamushi* in a new genus, *Orientia* gen. nov., as *Orientia tsutsugamushi* comb. nov. Int J Syst Bacteriol. 1995;45:589–91. 10.1099/00207713-45-3-589 8590688

[3] Watt G, Chouriyagune C, Ruangweerayud R, Watcharapichat P, Phulsuksombati D, Jongsakul K, Scrub typhus infections poorly responsive to antibiotics in northern Thailand. Lancet. 1996;348:86–9. 10.1016/S0140-6736(96)02501-9 8676722

[4] Bureau of Epidemiology, Department of Disease Control, Ministry of Public Health, Thailand. Annual epidemiologic surveillance report 2007. Scrub typhus [cited 2013 Mar 6]. http://www.boe.moph.go.th/Annual/ANNUAL2550/Part1/Annual_MenuPart1.html

[5] Bozeman FM, Elisberg BL. Serological diagnosis of scrub typhus by indirect immunofluorescence. Proc Soc Exp Biol Med. 1963;112:568–73 . 14014756 10.3181/00379727-112-28107

[6] Brown GW, Shirai A, Rogers C, Groves M. Diagnostic criteria for scrub typhus: probability values for immunofluorescent antibody and Proteus OXK agglutination titers. Am J Trop Med Hyg. 1983;32:1101–7 . 6414321 10.4269/ajtmh.1983.32.1101

[7] Enatsu T, Urakami H, Tamura A. Phylogenetic analysis of *Orientia tsutsugamushi* strains based on the sequence homologies of 56-kDa type-specific antigen genes. FEMS Microbiol Lett. 1999;180:163–9. 10.1111/j.1574-6968.1999.tb08791.x 10556707

[8] Swofford DL. PAUP*: Phylogenetic analysis using parsimony (and other methods) 4.0 beta 10. Sunderland (MA): Sinauer Associates. 2002.

[9] Tamura A, Takahashi K, Tsuruhara T, Urakami H, Miyamura S, Sekikawa H, Isolation of *Rickettsia tsutsugamushi* antigenically different from Kato, Karp, and Gilliam strains from patients. Microbiol Immunol. 1984;28:873–82 . 6438448 10.1111/j.1348-0421.1984.tb00743.x

[10] Tay ST, Rohani YM, Ho TM, Shamala D. Sequence analysis of the hypervariable regions of the 56 kDa immunodominant protein genes of *Orientia tsutsugamushi* strains in Malaysia. Microbiol Immunol. 2005;49:67–71 . 15665455 10.1111/j.1348-0421.2005.tb03641.x

[11] Ruang-Areerate T, Jeamwattanalert P, Rodkvamtook W, Richards AL, Sunyakumthorn P, Gaywee J. Genotype diversity and distribution of *Orientia tsutsugamushi* causing scrub typhus in Thailand. J Clin Microbiol. 2011;49:2584–9. 10.1128/JCM.00355-11 21593255 PMC3147819

[12] Traub R, Wisseman CL Jr, Jones MR, O’Keefe JJ. The acquisition of *Rickettsia tsutsugamushi* by chiggers (trombiculid mites) during the feeding process. Ann N Y Acad Sci. 1975;266:91–114. 10.1111/j.1749-6632.1975.tb35091.x 829479

[13] Walker JS, Gan E, Chye CT, Muul I. Involvement of small mammals in the transmission of scrub typhus in Malaysia: isolation and serological evidence. Trans R Soc Trop Med Hyg. 1973;67:838–45 . 10.1016/0035-9203(73)90012-6 4207572

[14] Takahashi M, Murata M, Hori E, Tanaka H, Kawamura A Jr. Transmission of *Rickettsia tsutsugamushi* from *Apodemus speciosus*, a wild rodent, to larval trombiculid mites during the feeding process. Jpn J Exp Med. 1990;60:203–8 . 2127292

[15] Frances SP, Watcharapichat P, Phulsuksombati D, Tanskul P, Linthicum KJ. Seasonal occurrence of *Leptotrombidium deliense* (Acari: Trombiculidae) attached to sentinel rodents in an orchard near Bangkok, Thailand. J Med Entomol. 1999;36:869–74 . 10593093 10.1093/jmedent/36.6.869

