# Supplementary material for: Scrub Typhus Outbreak, Northern Thailand, 2006–2007
Source: Emerg Infect Dis. 2013 May;19(5):774–7. doi: 10.3201/eid1905.121445 (PMC3647508; doi:10.3201/eid1905.121445)
Supplement: Technical Appendix — Clinical manifestations and laboratory test results for 26 scrub typhus–infected children from Ban Pongyeang, Thailand, who were hospitalized during June 2006–May 2007. [file 12-1445-Techapp-s1.pdf]

Article DOI: http://dx.doi.org/10.3201/eid1905.121445

Scrub Typhus Outbreak, Northern Thailand, 2006–2007

Technical Appendix

Technical Appendix Table**.** Clinical manifestations and laboratory test results for 26 scrub typhus–infected children from Ban Pongyaeng, Thailand, who were hospitalized during June 2006–May 2007*

							Test results for *Orientia tsutsugamushi*				
Patient		Signs and symptoms					IFA, IgM, IgG titer		PCR		
no.	Sex, age	Fever	Eschar	Headache	Chill	Rash	First serum sample	Second serum sample	First blood sample	Second blood sample	Isolation
P01	F, 2 y	+	+	–	+	–	400, 400	800, 800	+	–	+ (PYH1)
P02	M, 4 y	+	+	+	+	–	200, 50	400, 400	+	–	–
P03	F, 8 y	+	+	+	+	–	400, 50	800, 800	+	–	–
P04	M, 9 y	+	+	+	+	–	800, 800	800, 800	+	–	+ (PYH4)
P05	F, 12 y	+	–	+	+	–	200, 100	400, 800	+	–	–
P06	F, 7 y	+	+	+	–	–	50, Neg	400, 400	+	+	–
P07	M, 11 m	+	+	–	+	–	1600, 1600	1600, 1600	+	+	–
P08	M, 2 y	+	+	–	+	–	50, 800	100, 1600	+	–	ND
P09	M, 5 y	+	+	–	+	+	Neg, Neg	50, 50	+	–	ND
P10	M, 1 y	+	+	–	–	+	Neg, Neg	Neg, Neg	+	–	ND
P11	F, 9 y	+	+	+	+	+	400, 800	400, 800	–	–	ND
P12	M, 12 y	+	–	+	+	–	Neg, Neg	Neg, Neg	+	ND	ND
P13	M, 3 y	+	–	–	–	–	Neg, Neg	100, 100	+	+	ND
P14	M, 13 y	+	–	+	+	–	1600, 1600	1600, 1600	+	–	ND
P15	M, 5 y	+	–	+	+	–	1600, 800	1600, 800	+	+	ND
P16	M, 1 y	+	+	–	+	–	400, 400	ND	–	ND	ND
P17	M, 9 y	+	+	+	+	–	50, 400	50, 400	+	–	ND
P18	M, 1 y	+	+	–	+	+	Neg, Neg	400, 400	–	–	ND
P19	M, 6 y	+	+	+	+	–	400, 800	3200, 3200	+	+	ND
P20	M, 7 y	+	+	+	+	+	50, 50	800, 400	+	–	ND
P21	M, 1 y	+	–	–	–	–	400, 200	1600, 800	+	+	ND
P22	F, 5 y	+	+	+	–	+	400, 400	ND	+	ND	ND
P23	F, 2 y	+	–	–	+	–	1600, 800	3200, 800	+	–	ND
P24	M, 6 y	+	+	+	+	–	200, 50	400, 50	+	ND	ND
P25	F, 1 y	+	+	–	–	–	1600, 200	ND	+	ND	ND
P26	M, 13 y	+	+	+	–	–	Neg, Neg	ND	+	ND	ND

*IFA, indirect fluorescence antibody assay; Neg, negative; ND, not done.
